# Measuring ERCC1 protein expression in cancer specimens: Validation of a novel antibody

**DOI:** 10.1038/srep04313

**Published:** 2014-03-07

**Authors:** David Hersi Smith, Anne-Marie Kanstrup Fiehn, Louise Fogh, Ib Jarle Christensen, Tine Plato Hansen, Jan Stenvang, Hans Jørgen Nielsen, Kirsten Vang Nielsen, Jane Preuss Hasselby, Nils Brünner, Sussie Steen Jensen

**Affiliations:** 1 R&D, Dako A/S, Produktionsvej 42, DK-2600 Glostrup, Denmark; 2 Section for Molecular Disease Biology, Institute of Veterinary Disease Biology, Faculty of Health and Medical Sciences, Strandboulevarden 49, DK-2100 Copenhagen Ø, Denmark; 3 Department of Pathology, Copenhagen University Hospital, Blegdamsvej 9, DK-2100 Copenhagen Ø, Denmark; 4 Finsen Laboratory, Rigshospitalet and Biotech Research and Innovation Centre (BRIC), University of Copenhagen, Copenhagen Biocenter, Ole Maaloevs Vej 5, building 3, 3rd floor, DK-2200 Copenhagen N, Denmark; 5 Department of Pathology, Odense University Hospital, Winslowparken 15, DK-5000 Odense C, Denmark; 6 Department of Surgical Gastroenterology 360, Hvidovre Hospital, Kettegård Allé 30, DK-2650 Hvidovre, Denmark; 7 Institute of Clinical Medicine, Faculty of Health and Medical Sciences, University of Copenhagen, Blegdamsvej 3B, DK-2200 Copenhagen N, Denmark; 8 Current address: Centre for Innovation and Research, Ole Maaloevs Vej 3, DK-2200 Copenhagen N, Denmark.

## Abstract

Platinum chemotherapy remains part of standard therapies in the management of a variety of cancers. Severe side effects and a high degree of resistance to platinum drugs have led numerous researchers to search for predictive biomarkers, which could aid in identifying patients that are the most likely to respond to therapy. The ERCC1-ERCC4 endonuclease plays a critical role in the repair of platinum-DNA damage and has widely been studied in relation to sensitivity to platinum chemotherapy. The standard method to evaluate ERCC1 protein expression is through the use of immunohistochemistry with monoclonal antibody 8F1, an antibody that was recently found to bind an unrelated protein. The present study determines the specificity of a novel antibody, monoclonal antibody 4F9, and presents a method to evaluate ERCC1 expression in colorectal tumor specimens. Using relevant cell lines as controls, the specificity of antibody 4F9 was tested by immunoblotting, immunohistochemistry and immunofluorescence. Scoring guidelines to aid in the evaluation of ERCC1 tumor expression were developed and evaluated in archival formalin-fixed paraffin embedded colorectal cancer specimens. Antibody 4F9 was found to be specific by all methods applied and it was possible to evaluate the ERCC1 expression in the majority (85%) of colorectal cancer tumor specimens.

The identification of molecular markers that can help guide treatment decisions in cancer is central to improving the therapeutic index of the current arsenal of chemotherapeutic drugs. Platinum drugs, such as cisplatin and oxaliplatin, are part of standard chemotherapy regimens in several cancer types, including non-small cell lung cancer (NSCLC) and colorectal cancer (CRC). CRC is one of the leading causes of cancer death, accounting for approximately 8% of total cancer deaths worldwide^1^. In metastatic CRC, response rates to oxaliplatin-based chemotherapy are as low as 34%^2^. Therefore, to improve upon patient survival and quality of life, the identification of a predictive biomarker profile for platinum-based chemotherapy is essential, so that only patients that are likely to respond receive platinum chemotherapy.

Oxaliplatin and other platinum compounds inhibit tumor cell proliferation and induce cell death due to the formation of intracellular platinum-DNA adducts. These adducts consist of platinum-DNA monoadducts, platinum-DNA intra- and interstrand crosslinks, as well as DNA-protein crosslinks^3^,^4^. Platinum-DNA monoadducts and intrastrand crosslinks can be processed and repaired by nucleotide excision repair (NER)^5^. Interstrand crosslinks (ICL) are repaired through the activation of ICL repair, which involves several repair systems, such as homologous recombination, translesion synthesis, as well as NER^6^. The ERCC1-ERCC4 (XPF) heterodimer plays a critical role as a structure-specific endonuclease involved in both NER and ICL repair^7^,^8^, making it an interesting target for study in relation to platinum sensitivity/resistance.

A possible link between ERCC1 and platinum sensitivity has been investigated in a variety of cancer types^9^,^10^,^11^,^12^,^13^,^14^,^15^,^16^. Several studies using immunohistochemistry (IHC) to evaluate ERCC1 protein expression have relied on the use of anti-ERCC1 monoclonal antibody clone 8F1. This particular antibody has been found to cross-react with an unrelated protein^17^,^18^ and recent evidence suggests altered antibody specificity^19^. Taken together, the evidence supporting or opposing the use of ERCC1 protein expression in tailoring chemotherapy cannot be relied upon. Novel anti-ERCC1 antibodies have recently been developed, including antibody 4F9^18^, but before these can be used, thorough validation is required.

Inspired by the approach undertaken by Bhagwat et al.^17^, the purpose of the current study was to validate anti-ERCC1 antibody 4F9, allowing us and others to investigate the role of ERCC1 in relation to platinum sensitivity/resistance. To validate this antibody, we evaluated the specificity of antibody 4F9 and examined its use in IHC, where the influence of pre-analytical factors such as tissue fixation duration was mapped. Finally, the antibody was used in tumor specimens from a stage III CRC cohort, where oxaliplatin remains part of standard treatments, to test its ability to determine varying levels of ERCC1 expression in patient tumors, and therefore also its ability to potentially aid in clinical decision making.

## Results

### Testing the specificity of antibody 4F9

**Figure 1:**
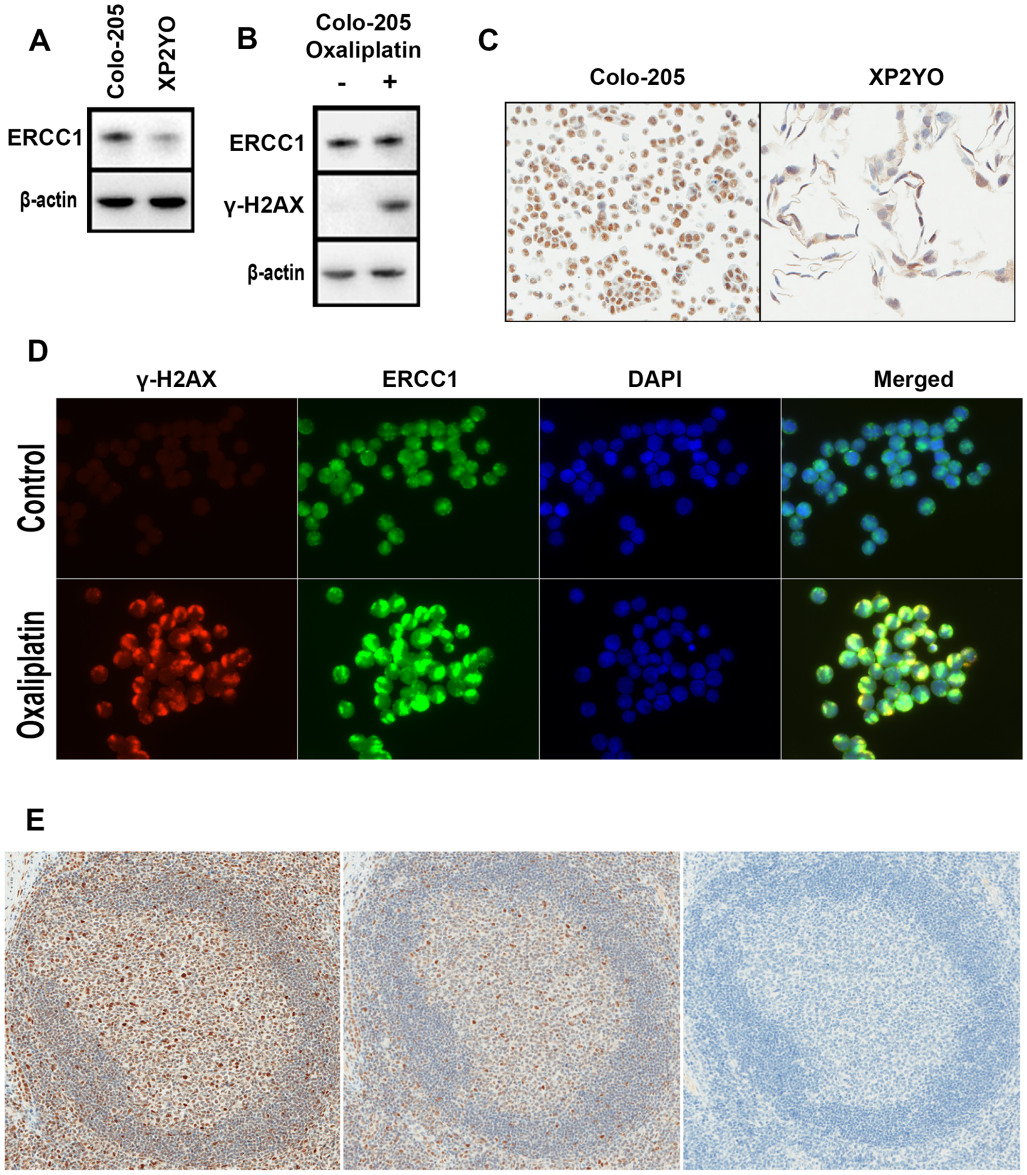
Testing the specificity of anti-ERCC1 antibody 4F9. (A) Cropped western blot of protein lysates from Colo-205 and XP2YO. (B) Cropped western blot of protein lysates from Colo-205 with and without 8 μM oxaliplatin. (C) Immunohistochemical staining of paraffin-embedded Colo-205 and XP2YO cells. (D) Detection of ERCC1 and γ-H2AX in Colo-205 chamber slide cultures by immunofluorescence. (E) Immunohistochemical staining of parallel tonsil sections in presence and absence of peptide corresponding to the antibody 4F9 epitope. A germinal center in the absence (left) and presence of epitope peptide at 1 μgml^−1^ (middle) and 10 μgml^−1^ (right).

For the detection of a protein of interest, ensuring antibody specificity is of paramount importance. The specificity of antibody 4F9 was assayed by western blot in protein lysates from cell lines Colo-205 and XP2YO. Antibody 4F9 produced a single and strong band corresponding to the molecular weight of ERCC1 (approximately 37 to 38 kDa) in Colo-205, whereas a relatively weak band was observed in XP2YO (Fig. 1A). In paraffin-embedded Colo-205 and XP2YO cells stained with antibody 4F9, strong nuclear expression of ERCC1 was observed in Colo-205 (see Fig. 1C). In XP2YO, weak cytoplasmic staining was observed, along with an absence of expression in the majority of nuclei.

To further evaluate the specificity of antibody 4F9, the detection of ERCC1 at sites of oxaliplatin-induced DNA damage was assayed by immunofluorescence in Colo-205 cells. Untreated cells showed little or no γ-H2AX signal generation and relatively uniform ERCC1 expression in nuclei (Fig. 1D). In oxaliplatin-treated cells, large γ-H2AX foci were observed with similarly large co-localized ERCC1 foci. Western blot of Colo-205 lysates from treated and untreated cultures (Fig. 1B) confirmed the presence of γ-H2AX in oxaliplatin-treated cells only.

To confirm that antibody 4F9 reacts with the reported epitope, a peptide containing the epitope (AEYAISQ) was constructed and pre-incubated with antibody 4F9 prior to IHC staining. As shown in Fig. 1E, without the peptide, antibody 4F9 stained tonsil germinal center cells in a variety of intensities. Upon inhibition with the peptide at 1 μgml^−1^, the observed staining intensities decreased substantially. Complete inhibition was achieved with 10 μgml^−1^ peptide, where no staining was observed. Antibody 4F9 was also tested by an enzyme-linked immunosorbent assay (ELISA) against a random peptide or a peptide representing the 4F9 epitope (see supplementary note 4). Above-background absorbance was only detected in wells containing the 4F9 epitope (see fig. S2).

### Identification of references and effect of fixation

To evaluate the expression levels of ERCC1 in tumor cells, where the observed staining intensity may be affected by pre-analytical and analytical factors, we sought to identify relevant tissues that express the protein, so that these tissues could be used as an external reference. Tonsil tissue appeared to stain in an easily recognizable pattern (see Fig. 1E) and was selected as an external reference. A single tonsil specimen was studied and staining intensities of various cell populations were graded on a scale from 0 to 3 corresponding to none (0), low (1), moderate (2) and high (3) staining intensity by two investigators. Fibroblasts scored 0 and 1, whereas endothelial cells scored 0, 1 and 2. The mantle zone surrounding the germinal center scored predominately 0 and 1, while the germinal center generally stained 1 and 2, unidentified cell populations (consisting of numerous cell types, including macrophages and centroblasts) scattered throughout the germinal center scored 3 (see Fig. 1E). The squamous epithelium harbored cells of all staining intensities, with stronger intensities observed closer to the basal layer. These observations did not vary among tonsils tested (see supplementary note 3).

**Figure 2:**
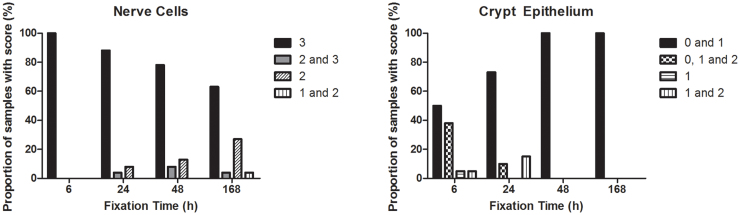
Graphical representations of results from fixation study. Comparisons between selected cell populations with an external tonsil reference.

**Figure 3:**
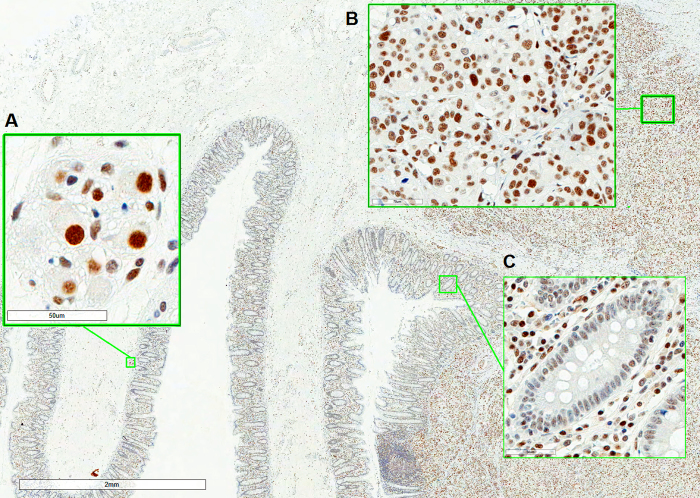
Immunohistochemical staining of a stage III CRC tumor specimen. (A): Ganglion cells. (B): Tumor cells. (C): Crypt epithelium. The applied scoring guidelines require a comparison of tumor cells (B) with ganglion cells (strongest intensity is reference for score 3) (A) and crypt epithelium (weakest intensity is reference for score 1) (C). The tumor consists of cells producing staining intensities similar to ganglion cells, as well as cells that stain weaker, but stronger than crypt epithelium (i.e. moderate). Therefore, the final score of this tumor was 2–3, which was reported as ‘ERCC1 high – homogenous'.

To identify relevant internal IHC scoring references in colorectal tissue, numerous tissue specimens were stained and screened for easily recognizable non-cancerous cells, which appeared to stain similarly across samples (data not shown). This screening resulted in the identification of parasympathetic ganglion cells (Meissner and Auerbach's plexuses) and epithelial cells lining the colorectal crypts as potential references. Ganglion cells generally showed high intensity ERCC1-specific staining, whereas crypt epithelial cells exhibited relatively low intensity staining. To determine whether the staining intensity of these cell types was affected by fixation time, small sections from 19 different tumors were allocated to receive 6, 24, 48 and 168 h fixation in NBF (CRC fixation time study), resulting in a total of 228 sections. These sections were stained and compared to a tonsil tissue reference (Fig. S1). Ganglion cells were present in 94 tissue sections from a total of 228 (41.2%). A median of 5 observations were made per tumor (range: 1–10) with a median of 23.5 observations per fixation time (range: 23–25). As shown in Fig. 2, the staining intensity of these cells decreased at longer fixation times, specifically with a decreasing proportion of samples harboring ganglion cells with an intensity score of 3 at longer fixation times (100% and 6 h, 89.0% at 24 h, 78.2% at 48 h and 63.6% at 168 h). A test of association between the staining intensity of ganglion cells and tissue fixation time revealed a significant interaction (Cochran-Mantel-Haenzel test, p = 0.002, after adjusting for which of the original 19 tumors the section stemmed from). The highest staining intensity observed in ganglion cells was therefore chosen as an internal reference for the high (3) ERCC1 expression category (see Fig. 3).

The colorectal crypt epithelium was present in 65 (28.5%) tissue sections. A median of 4 observations per tumor (range: 0–6) and 16.5 observations per fixation time (range: 13–19) were made. The majority of cells scored as follows: 0 and 1 (50.0%) or 0, 1 and 2 (38.9%) following 6 h fixation (see Fig. 2). The staining intensity of these cells decreased at longer fixation times, specifically an increasing fraction of crypt epithelial cells scoring 0 and 1 were observed (73.7% at 24 h, 100% at 48 h and 168 h). A test of association between the staining intensity of these cells and tissue fixation time revealed a significant interaction (Cochran-Mantel-Haenzel test, p = 0.007, after adjusting for tumor of origin). The weakest positive staining intensity observed in crypt epithelial cells was therefore chosen to reflect the low (1) ERCC1 expression category (Fig. 3).

### Formulation of scoring system and training study

To determine the feasibility of using the previously identified internal references to assign staining intensities in CRC tumor samples, 50 FFPE CRC tumor samples were stained and studied (referred to as training study, see table 1). Ganglion cells were observed in 92% (46/50) of specimens. The majority of ganglion cells stained with either high (3), or moderate and high (2 and 3) intensity relative to relevant cells in a tonsil reference. Colorectal crypt epithelium was present in 96% (48/50) of samples and mostly contained cells with no or low intensity staining (see table S3). Both references were present in 90% (45/50) of samples.

Prior to final formulation of the IHC scoring system, intrasection tumor heterogeneity was investigated. To allow for limited fluctuation in ERCC1 expression, homogeneity was defined as the difference in staining intensities being no greater than ‘1'.

**Figure 4:**
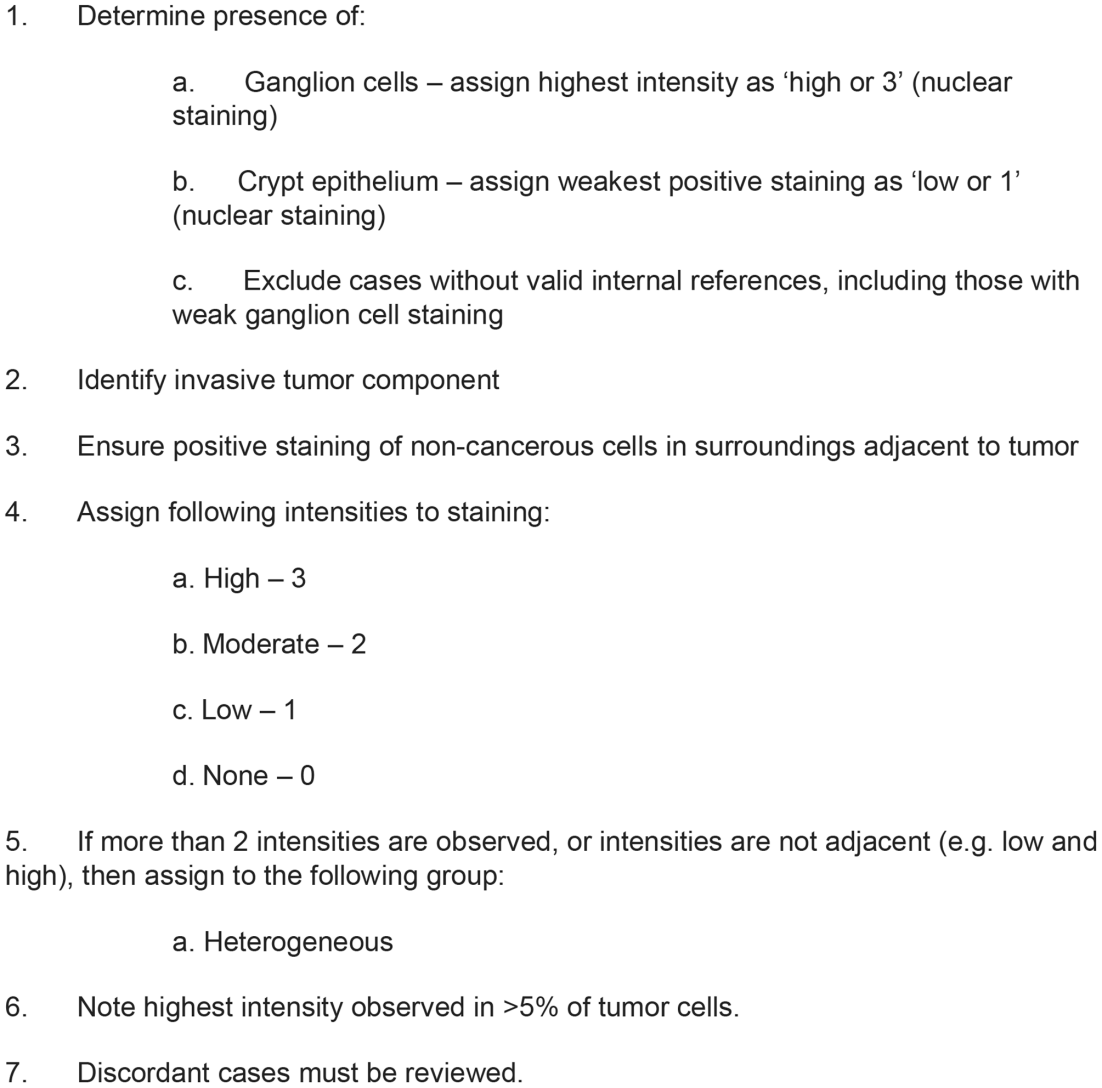
ERCC1 IHC Scoring System.

By this definition, 11.1% (5/45) of samples were categorized as heterogeneous. Two patterns of heterogeneity were observed. The first pattern was defined as heterogeneity present in the entire tumor section, whereas the second pattern was due to the presence of two distinct tumor components, indicating multiple clones. Regardless of type, the highest staining intensity observed in >5% tumor cells was noted for each tumor, as well as whether the expression was homogenous or heterogeneous in tumor cells (see fig. 4 for final formulation of scoring system). Scoring of tumor cells by using the aforementioned internal references categorized the majority (71.1%) of tumor specimens as low (1) (see Table 2). As shown in Table 1, ERCC1 expression was not associated with tumor stage. ERCC1 expression was not significantly associated with tumor localization (Fisher's test, p = 0.06), although 10 of 11 (90.9%) higher scoring (scores: 2 and 3) tumors originated from the colon.

### ERCC1 expression in pilot study

To investigate whether antibody 4F9 could potentially be used in a clinical decision making, a pilot study was performed by evaluating ERCC1 expression in tumor specimens from a stage III CRC cohort. At this disease stage (stage III), oxaliplatin-based chemotherapy is part of standard regimens. A total of 141 tumor specimens were stained. Internal references were absent in 9 samples (6.4%) and in 12 samples (8.5%) staining was too weak, indicative of extensive fixation. Immunostaining and scoring was therefore successful for 120 samples (85.1%). Initially, the two observers were in agreement in 97 of 120 cases (or 80.3%, weighted kappa = 0.75, 95% CI: 0.66–0.84). The majority of discordant cases (13 out of 23 cases - 56.5%) were scored as 3 by at least one observer. When staining intensity was considered as a binary variable (0, 1 versus 2, 3), interobserver agreement was 91.7% (110 of 120 cases, kappa = 0.83, 95% CI: 0.73–0.93). As shown in Table 2, the majority of samples could be categorized as ERCC1 low (46.7%) or ERCC1 moderate (35.0%) (see Fig. 3 for an example). The median value of ERCC1 IHC scores was 1 in this cohort. ERCC1 expression was not significantly associated with patient age, gender or tumor location (see Table 1). Tumor heterogeneity was observed in 21 (17.5%) specimens.

## Discussion

### Specificity

The predictive value of ERCC1 expression in NSCLC was initially demonstrated in a study conducted by Olaussen and colleagues^9^. Patients with ERCC1-negative tumors appeared to benefit from adjuvant cisplatin-based chemotherapy, whereas those with ERCC1-positive tumors gained no benefit. In this initial study, antibody 8F1 appeared to bind ERCC1 specifically^27^. Subsequent research revealed that this antibody binds ERCC1, as well as an unrelated protein, namely PCYT1A^18^,^17^. Efforts to reproduce these initial findings using the original specimens have failed^19^. For that reason, we hypothesize that at some time between the original NSCLC study in 2006^9^ and the first report of cross-reactivity of 8F1 in 2009^17^, the specificity of antibody 8F1 was altered. Although these results are published, antibody 8F1 remains the most widely used anti-ERCC1 to date with continued use in 2013^28^,^29^,^30^. This use continues to shed uncertainty on published findings related to ERCC1 and highlights the need for a novel ERCC1-specific antibody.

To ensure that antibody 4F9 is specific, a three pronged approach was undertaken in the present study: firstly, specificity for size and location was tested in immunoblots of lysates from Colo-205 and XP2YO. XP2YO was selected as a control for low ERCC1 expression as cell lines without any functional ERCC1 are very difficult to maintain in culture^31^. XP2YO harbors several mutations in the *ERCC4* gene, which cause ERCC4 expression to be particularly low in this cell line^17^,^32^,^33^. Interestingly, ERCC1 is rapidly degraded unless the protein is in complex with ERCC4^34^, making XP2YO a relevant choice as a low expression control for ERCC1-specific antibodies. In the current study, antibody 4F9 detected substantial ERCC1 in Colo-205, while ERCC1 was detected at markedly lower levels in XP2YO. Secondly, specificity for functionality (ERCC1 DNA repair activity) and localization was tested by immunofluorescence. Due to its involvement in the processing and removal of DNA damage, the ERCC1-ERCC4 heterodimer should co-localize to sites of platinum-induced double strand DNA breaks, measured here by γ-H2AX phosphorylation^8^. In the present study, antibody 4F9 correctly generated signals for ERCC1 at double strand DNA breaks, indicating specificity and supporting the use of antibody 4F9 in biomarker investigations related to oxaliplatin. Finally, to confirm the specificity of antibody 4F9 to its reported epitope, the interaction between antibody and peptide was tested by IHC inhibition and ELISA. This revealed that antibody 4F9 binds this epitope in a concentration-dependent manner. These results are in line with those identified by Friboulet and colleagues^19^. It should be noted that this amino acid sequence is present in ERCC1 isoforms 201, 202, 203, but not 204. Taken together, these findings suggest that antibody 4F9 binds ERCC1 specifically, a result which is in line with those previously reported^18^.

### Scoring guidelines

During tissue fixation, formalin reacts with a variety of biological end groups, forming hydroxymethyl adducts. These adducts react with other end groups, forming (methylene) crosslinks between proteins, thereby preserving protein localization and tissue morphology, as well as inactivating factors that may cause tissue degradation (reviewed in^35^). Crosslink formation is reversible by antigen retrieving tissue pretreatment. The extent of crosslink formation is related to tissue fixation time, which is often unknown in archival material and practices may vary from institution to institution, making it a pre-analytical factor which can be highly variable in archival material from multicenter studies. If antibody 4F9 is to be used in IHC, the effects of fixation time must be elucidated and practical scoring guidelines must be created.

When subjected to longer fixation periods, the ERCC1-specific staining intensity of particular cell populations in colorectal tissue appeared to decrease significantly. This finding raised the requirement of identifying relevant internal staining references, which could compensate for the fixation-dependent decrease in the observed ERCC1-specific staining intensity. Ganglion cells and cells of the crypt epithelium stained with higher intensities following 6 h fixation than at longer fixation periods. While fixation time may vary day-to-day and institution-to-institution, we hypothesized that 24 to 48 h was most representative fixation period for archival tumor material. Interestingly, this fixation time-dependence does not appear to only affect 4F9 staining, but also that other anti-ERCC1 antibodies, such as 8F1, 8K105, 2E12, FL297 and SMP243 (unpublished data). It should be noted that additional factors, such as tissue ischemia time and tumor volume, may also influence the staining intensity observed by IHC, but these were not investigated. Using the findings of the fixation study, we chose to select ganglion cells (highest intensity) and the crypt epithelium (weakest positive intensity) as references for the high (3) and low (1) ERCC1 expression categories, respectively (see Fig. 3). The biological rationale for relatively high ERCC1 expression in ganglion cells may be related to protection from DNA damage^36^. It is unknown whether the expression of ERCC1 in colorectal crypts is affected by exogenous factors. It should be noted that similar staining intensities were observed in both the 19 tumor fixation material and the training study, suggesting limited exogenous influence on ERCC1 expression. Through the use of these references, we have designed a dynamic scoring system which can compensate for a variety of tissue fixation times, but optimized for 24 to 48 h fixation.

We have in the current study chosen to record the relative expression level of tumor cells without accounting for the proportion of tumors cells with each expression level, e.g. by a semi-quantitative H-score. This decision was made to ensure that the scoring system is both practical and simple. Furthermore, we hypothesized that even a minor component of tumor cells with high ERCC1 expression may form the basis for tumor recurrence following oxaliplatin-based chemotherapy. This hypothesis can only be tested in material from patients that have undergone relevant chemotherapy and therefore the adaptation of an H-score may be appropriate to investigate a predictive effect of ERCC1 expression in relation to oxaliplatin-based chemotherapy.

In the present study, a scoring system was developed to evaluate ERCC1 expression in full sections from CRC tumors. The selection of colorectal crypt epithelial and ganglion cells as internal references limits the usability of the scoring system in specimens that do not contain these cell types, such as biopsies. Therefore, efforts to develop biopsy-specific references, or valid external controls, are warranted.

### ERCC1 tumor expression

To determine whether antibody 4F9 could be used to distinguish between different levels of ERCC1 expression in archival material, CRC tumor specimens from two cohorts were evaluated. These were selected because they are representative of patients that would undergo platinum-based chemotherapy today. Tissue references were present in the majority of samples from both CRC cohorts, and the staining intensity was acceptable in 100 and 91.5% in cohorts 1 and 2, respectively. In both cohorts, the largest fraction of tumors expressed low levels of ERCC1. Interestingly, a higher percentage of tumors scored 2 and 3 in the pilot study cohort (48.3%) when compared to the training cohort (24.4%). This finding could not be attributed due to higher tumor stage, as no association was found between protein expression and patient clinicopathological characteristics in the training study (see Table 1).

In the pilot study, interobserver agreement was acceptable (80.3%). Interestingly, the major source of disagreement was related to the high expression category (score 3). This disagreement could be circumvented by applying a binarized version of the scoring system, where tumor specimens can be assigned to either a positive (scores 2, 3) or negative (scores 0, 1) ERCC1 expression level category. While this improves interobserver agreement in the present study, it also limits the resolution of the scoring system in detecting differences in ERCC1 expression. A higher resolution may be crucial in future investigations into the possible link between ERCC1 expression and patient outcome following platinum-based chemotherapy.

Recent evidence suggests that only ERCC1 isoform 202 is responsible for the removal of cisplatin-adducts^19^. Due to a high degree of homology between isoforms, it is not possible to produce antibodies specific for only the 202 isoform. Therefore, whether ERCC1 expression, specifically isoforms 201, 202 and 203, is related to outcome following platinum-based chemotherapy remains to be studied. It should be noted that low ERCC1 mRNA expression (all isoforms) has previously been shown to be linked to benefit from oxaliplatin in advanced CRC^10^,^19^. Furthermore, clarification of the exact roles of each of the four ERCC1 isoforms in the repair oxaliplatin-induced DNA damage in CRC is required. We plan on investigating ERCC1 as a predictive biomarker for likely outcome following oxaliplatin-based chemotherapy in metastatic CRC through the use of antibody 4F9 in IHC, as well as by fluorescent in situ hybridization. We have previously studied *ERCC1* gene copy number alterations in tumor specimens from the patient of the pilot study^37^. There appears to be a significant correlation between *ERCC1* gene copy number and ERCC1 protein expression (see supplementary note 1, table S4), suggesting that this may be in fact be the underlying mechanism for ERCC1 overexpression. With both these assays, we hope to shed light on the link between ERCC1 and platinum-based chemotherapy.

In conclusion, the current study highlights the necessity of antibody validation before investigational use in archival tumor material. While antibody 4F9 was found to be specific, a fixation time-dependent decrease in staining intensity was observed in IHC. To counter this, CRC-specific internal IHC staining references were identified and successfully applied. Taken together, the validation of antibody 4F9 will allow us and others to confidently investigate the effects of ERCC1 protein expression in relation to patient outcome following oxaliplatin-based chemotherapy in CRC.

## Methods

### Cell lines

The CRC cell line Colo-205 was obtained from the NCI/Development Therapeutics Program and was propagated in complete media: RPMI 1640 (Invitrogen) with 10% fetal bovine serum (Invitrogen). The transformed ERCC1-deficient human fibroblast cell line XP2YO, an immortalized fibroblast cell line stemming from a xeroderma pigmentosum complementation group F patient^20^, was obtained from Coriell Cell Repositories and was cultured in complete media: DMEM (Invitrogen) with 10% fetal bovine serum (Invitrogen).

### Patient material

#### Tonsils

A total of six fresh tonsils were collected at the Department of Pathology, Ålborg University. Tonsils were trimmed and cut into two sections. Each section was allocated to receive either 6 or 30 hour fixation in 10% neutral buffered formalin (NBF). All tonsil specimens were completely anonymized prior to receipt. According to the Danish law on the Research Ethics Committee System and handling of biomedical research, as well as communication between Dako and the Danish Committee on Biomedical Research Ethics and the Regional Ethics Committee (IRB), the tests performed on tonsil specimens are not subject to an approval by the IRB system because such studies are considered quality control projects. Therefore, no IRB approval was obtained for the use of these specimens.

#### CRC tissue fixation study

A total of 19 fresh resected primary CRC tumors were collected at the Department of Pathology, Odense University Hospital between August 1995 and October 1996 (described in detail in^21^). Briefly, tumors were trimmed for normal intestinal tissue and cut into parallel sections. For each tumor, two sections were chosen by random systematic sampling and cut into approximately 2 mm parallel tissue chips, which were subsequently allocated to receive approximately 6, 24, 48 and 168 hours (1 week) fixation in NBF. Following fixation, tissue chips were embedded in paraffin and new paraffin blocks were created containing three tissue chips for each fixation time and tumor, resulting in two blocks per tumor. Tumor specimens were completely anonymized prior to receipt. Similar to tonsil specimens, this work can be considered a quality control project. Therefore, no IRB approval was obtained for the use of these specimens.

#### CRC cohort 1: training study

Histologically proven stage I to III CRC specimens for the training study were acquired from the Colorectal Cancer Follow-up (C-FUP) study^22^. The C-FUP trial included 597 patients intended curative surgery for CRC from January 1983 to June 1994. Fifty primary tumor specimens were randomly selected from patients enrolled in group 1 (see^22^). The C-FUP trial was conducted in accordance to Helsinki II Declaration and was approved by the Regional Ethical Committee for the Counties of Vejle and Funen and the Danish Data Protection Agency. Informed consent was obtained from all subjects. The exact duration of fixation was not registered, but ranged from 12 hours to 1 week.

#### CRC cohort 2: pilot study

A total of 154 patients with histologically proven stage III adenocarcinomas were selected as previously described^23^,^24^,^25^. FFPE primary tumor specimens containing sufficient tumor cells were available for 141 (91.6%) patients. As part of the RANX05 trial, patients were randomized to receive Ranitidine or placebo for up to five years, as adjuvant chemo- and/or radiotherapy as this was not part of the standard CRC treatment in Denmark at the time (1991–1993). Ranitidine had no effect on survival^26^. All participants provided written informed consent. The RANX05 trial was conducted in accordance with the Helsinki II Declaration and was approved by the Danish National Board of Health, Data Protection Agency and Central National Ethics Committee.

### Immunoblotting

Cells were cultured in the presence and absence of oxaliplatin (Fresenius Kabi) at a final concentration at 8 μM for 24 hours (see supplementary note 2). Ten μg total protein from cell lines Colo-205 and XP2YO were boiled (5 min) in 6× loading buffer. Anti-ERCC1 antibody antibody 4F9 (Dako) was diluted 1:500 (1.0 μgml^−1^). Anti-γ-H2AX (Clone PA1-25001, Thermo Scientific), detecting histone H2AX phosphorylated at serine 139 (a marker for DNA double strand breaks), was diluted 1:500. As a protein loading control, anti-β-actin (clone AC-15, Abcam) was applied at a dilution of 1:1000. For visualization, the Amersham ECL Select Western Blotting Detection Reagent kit (GE Healthcare) was used.

### Immunofluorescence

For immunofluorescence, cells were seeded in chamber slides (Nunc) and allowed to plate for 72–96 h. Cells were then treated with or without oxaliplatin (Fresenius Kabi) at a final concentration at 8 μM for 24 h (see supplementary note 2). Staining was performed using an Autostainer instrument (Dako). Briefly, cells were permeabilized by incubation with PBS containing 0.25% Triton X-100 (10 min) and thereafter blocked with 10% goat serum in PBS for 1 hour. Slides were treated with peroxidase block (5 min), and then incubated with a polyclonal anti-γ-H2AX rabbit antibody (dilution 1:1200) for 20 min to detect DNA double strand breaks. Signal was generated by use of goat anti-rabbit secondary antibody coupled to HRP (20 min) (Dako) with a Texas Red-based fluorescent tyramide substrate (3 min) (Dako). To terminate signal generation, slides were treated with peroxidase block. Slides were subsequently incubated with anti-ERCC1 antibody 4F9 at 1.66 μgml^−1^ (corresponding to a dilution of 1:300) for 20 min. Antibody 4F9 was visualized by use of goat anti-mouse secondary antibody coupled to HRP with a FITC-based fluorescent tyramide substrate (3 min). Finally, slides were washed and counterstained with DAPI and mounted.

### Immunohistochemistry

#### Immunohistochemical staining

For immunostaining of paraffin embedded cell lines (see supplementary note 2) and FFPE patient specimens, all reagents came from the EnVision™ FLEX, High pH kit (K8010) (Dako). Antigen retrieval was performed at pH 9 for 20 min using a PT Link module (Dako). Staining was performed in an Autostainer instrument (Dako) or manually according to manufacturer's instructions. Briefly, slides were treated with peroxidase block (5 min), then incubated with anti-ERCC1 antibody 4F9 at 1.66 μgml^−1^ for 20 min. Visualization of antibody binding was done by incubation with a labeled HRP-polymer for 20 min and signal was generated with a 3.3′-diaminabenzidin (DAB) chromogen (10 min). Hematoxylin was used for counterstaining.

#### IHC inhibition

A peptide containing the antibody 4F9 epitope (AEYAISQ, sequence provided by Origene) (PolyPeptide Laboratories) was pre-incubated with antibody 4F9 at varying concentrations (overnight, 4°C). Slides were subsequently stained as previously described.

#### Microscopical analysis

Tonsil and CRC fixation specimens were evaluated by two investigators (AMK and JPH) simultaneously. Only nuclear ERCC1 staining was considered. Only the nuclear staining intensity of relevant cell types was recorded by comparison to a tonsil reference. Training study CRC specimens were scored by both investigators individually. Discordant cases were reviewed and agreement was reached. For the stage III CRC material, both investigators were unaware of clinical data. Cases without internal references or extremely weak staining of references were excluded.

### Statistics

SAS versions 9.1 and 9.2 (SAS Institute, Cary, USA) were used to perform all statistics. The association between fixation time and staining intensity of relevant cell types was investigated by use of the Cochran-Mantel-Haenzel test, adjusting for tumor of origin. Interobserver agreement was measured by concordance (calculated by concordant cases/total cases) and by kappa statistics. Association between ERCC1 expression and clinicopathological characteristics was investigated by Fisher's test. All calculated p-values were considered significant at 0.05.

## Author Contributions

Design of experiments: D.H.S., J.P.H., N.B., K.V.N., S.S.J., J.S., T.P.H. Conducted in vitro experiments: D.H.S., L.F. Scoring of tissue specimens: A.K.F., J.P.H. Statistical analysis: D.H.S., I.J.C. Contributed reagents: H.J.N., S.S.J. All authors reviewed the manuscript.

## Supplementary Material

Supplementary Information Supplementary Information

## Figures and Tables

**Table 1:** Clinicopathlogic characteristics of patients included in the present study

Patient Cohort			Frequency (%)	Association with ERCC1 expression
**Training study**	**Stage**	**I**	7 (15.6)	0.4^a^
		**II**	25 (55.6)	
		**III**	13 (28.9)	
	**Location**	**Colon**	29 (64.4)	0.06^a^
		**Rectum**	16 (35.6)	
				
**Pilot study**			**Median (range)**	
	**Age**		68 (33–85)	−0.08, p = 0.38^b^
			**Frequency (%)**	
	**Gender**	**Male**	73 (60.8)	0.30^a^
		**Female**	47 (39.2)	
	**Stage**	**III**	120 (100)	-
	**Location**	**Colon**	62 (51.7)	0.15^a^
		**Rectum**	58 (48.3)	

^a^p-value from fisher's exact test.

^b^spearman's rank correlation.

**Table 2:** ERCC1 expression in the two cohorts analyzed in the present study

IHC Score	Frequency (%)	
	Training study	Pilot study
0	2 (4.4)	6 (5.00)
1	32 (71.1)	56 (46.7)
2	8 (17.8)	42 (35.0)
3	3 (6.7)	16 (13.3)
