# Supplementary material for: Measuring ERCC1 protein expression in cancer specimens: Validation of a novel antibody
Source: Sci Rep. 2014 Mar 7;4:4313. doi: 10.1038/srep04313 (PMC3945488; doi:10.1038/srep04313)
Supplement: Supplementary Information [file srep04313-s1.pdf]

Supplementary Information

Title: **Measuring ERCC1 protein expression in cancer specimens: Validation of a novel antibody**

Authors: David Hersi Smith [a,b], Anne-Marie Kanstrup Fiehn[c], Louise Fogh [b], Ib Jarle Christensen [d], Tine Plato Hansen [e], Jan Stenvang [b], Hans Jørgen Nielsen [f,g], Kirsten Vang Nielsen [a,1], Jane Preuss Hasselby [c], Nils Brünner [b], Sussie Steen Jensen [a]

Affiliations:

[a] R&D, Dako A/S, Produktionsvej 42, DK-2600 Glostrup, Denmark

[b] Section for Molecular Disease Biology, Institute of Veterinary Disease Biology, Faculty of Health and Medical Sciences, Strandboulevarden 49, DK-2100 Copenhagen Ø, Denmark

[c] Department of Pathology, Copenhagen University Hospital, Blegdamsvej 9, DK-2100 Copenhagen Ø, Denmark

[d] Finsen Laboratory, Rigshospitalet and Biotech Research and Innovation Centre (BRIC), University of Copenhagen, Copenhagen Biocenter, Ole Maaloevs Vej 5, building 3, 3rd floor, DK-2200 Copenhagen N, Denmark

[e] Department of Pathology, Odense University Hospital, Winslowparken 15, DK-5000 Odense C, Denmark.

[f] Department of Surgical Gastroenterology 360, Hvidovre Hospital, Kettegård Allé 30, DK-2650 Hvidovre, Denmark

[g] Institute of Clinical Medicine , Faculty of Health and Medical Sciences, University of Copenhagen, Blegdamsvej 3B, DK-2200 Copenhagen N, Denmark

[1] Current address: Centre for Innovation and Research, Ole Maaloevs Vej 3, DK-2200 Copenhagen N, Denmark

Contents

Supplementary Note 1: Correlation to *ERCC1* gene copy number 3
Supplementary Note 2: Sample preparation 4
Sample preparation for immunoblotting 4
Sample preparation of cell lines for immunofluorescence 4
Sample preparation of cell lines for immunohistochemistry 4
Supplementary Note 3: Tonsil Fixation Experiments 5
Supplementary Figures 6
Supplementary Tables 8

Supplementary Note 1: Correlation to *ERCC1* gene copy number

To investigate whether *ERCC1* gene copy number alterations affect ERCC1 protein expression, ERCC1 IHC scores were compared with previously acquired *ERCC1* fluorescent in situ hybridization (FISH) data from this cohort ([1]). FISH data was available for 119 (99.2%) patients. The *ERCC1* gene probe was used in combination with a centromere 2 (CEN-2)-specific probe, which is used as an estimator of cellular ploidy levels (i.e. total number of chromosomes) [1]. Linear regression analysis revealed a significant relationship between *ERCC1* gene copy number and IHC score (parameter estimate: 0.15, 95% CI: 0.03-0.27, p=0.013). A relationship between the *ERCC1/*CEN-2 ratio and ERCC1 protein expression was also observed (parameter estimate: 0.11, 95% CI: 0.03-0.18, p=0.005).

Using a *ERCC1*/CEN-2 cut-off of ≥ 1.5 (representing a 50% increase in gene copy number relative to cellular ploidy, or an additional copy of the gene in a diploid cell), a total of 34 (28.6%) specimens could be identified as harboring a gene gain. When compared to ERCC1 protein expression (positive: score 2 and 3, negative: score 0 and 1), a total of 22 (64.7%) tumors were positive by both assays (see Table S4). A significant association between gene gain and higher protein expression was observed (Fisher's test, p=0.03). It should be noted that we have previously identified two patients harboring a deletion of the ERCC1 gene (*ERCC1*/CEN-2 < 0.8) in this cohort. These samples scored 0 and 1 in the current study.

It should be noted that while the *ERCC1* gene naturally encodes at all isoforms, we can only detect ERCC1 isoforms 201, 202 and 203 by IHC in the current study. In NSCLC tumor samples, these isoforms appear to be expressed heterogeneously, with some specimens expressing high levels of some isoform, but not others [2]. Interestingly, we have previously reported that *ERCC1* gene copy number alterations appear to be homogenous in tumors, suggesting that additional factors are involved in the regulation of ERCC1 expression [3].

Supplementary Note 2: Sample preparation

Sample preparation for immunoblotting

For protein extraction, 2.1×10^{6} cells were seeded in 75 cm^2 flasks and allowed to plate for 72-96 h. Lysates were harvested individually by rinsing the cells twice in ice cold PBS and lysed by incubation (5 min, room temperature) in Mammalian Protein Extraction Reagent (Themo Scientific) containing Pierce Protease and Phosphatase Inhibitor Tablets (Themo Scientific). Cells were centrifuged (14.000 g, 10 min, 4 °C) and each supernatant was transferred to a new tube. Protein concentration was determined by comparison to an albumin standard through the use of a BCA Protein Assay kit (Themo Scientific), which was performed according to manufacturer's instructions.

Sample preparation of cell lines for immunofluorescence

For immunofluorescence, cells were seeded with 20.000 cells/chamber in chamber slides (Nunc) and allowed to plate for 72-96h. Cells were then treated with oxaliplatin (Fresenius Kabi) at a final concentration at 8 µM for 24 h. Cells were washed twice in ice cold PBS and fixed in ice cold 10% PBS-buffered formalin for 30 min. Finally, fixed cells were washed in PBS and washed once in 70% ethanol.

Sample preparation of cell lines for immunohistochemistry

For paraffin embedment of cells, 4.2×10^{6} cells were seeded in 150 cm^2 flasks and allowed to plate for 48 h. Culture medium was changed and cells were harvested after additional 48 h. Cells were subsequently washed in ice cold PBS and fixed in ice cold 10 % PBS-buffered formalin (30 min.). Fixed cells were washed in PBS, mechanically detached and pipetted to a 15 ml tube. Tubes were centrifuged for (400g, 5 min.) and supernatant was removed. The cells were embedded in a drop of bactoagar and transferred to 70 % ethanol before paraffin embedment.

Supplementary Note 3: Tonsil Fixation Experiments

To determine whether staining intensities were affected by tissue fixation time, six paired tonsil specimens, fixed for 6 and 30h in NBF, were compared. Fibroblasts and germinal center cells stained with identical intensities at both fixation times. Staining of endothelial cells and the squamous epithelium showed little, but no-significant variation (McNemar's test, p= 0.5 and 1.0, respectively - see tables S1 and S2 for details). Therefore, the aforementioned tonsil cell populations were deemed suitable as an external IHC scoring control to be used as a reference to assign staining intensities (on a scale from 0 to 3) in other tissues.

Supplementary Note 4: Enzyme-Linked Immunosorbent Assay (ELISA)

For ELISA, 96-well plates (Nunc) were coated overnight with either the 4F9 peptide or a random peptide (sequence: CSFYLNRNPEEPNGGE) at 4 °C using a phosphate coating buffer. Plates were washed and antibody 4F9 was added at relevant concentrations and allowed to incubate for 2 h at room temperature. Plates were subsequently washed and secondary goat anti-mouse peroxidase-conjugated antibody (Dako) was added and incubated for 1 h at room temperature. A chromogenic substrate (TMB -3,3'-5,5' tetramethylbenzidine) (Dako) was added to wells and the reaction was stopped after 15 min. by the addition of 0.5M sulfuric acid. Results were read at 450 nm using an ELISA reader. Mean absorbance (from duplicates) was calculated for all data points.

Supplementary Figures

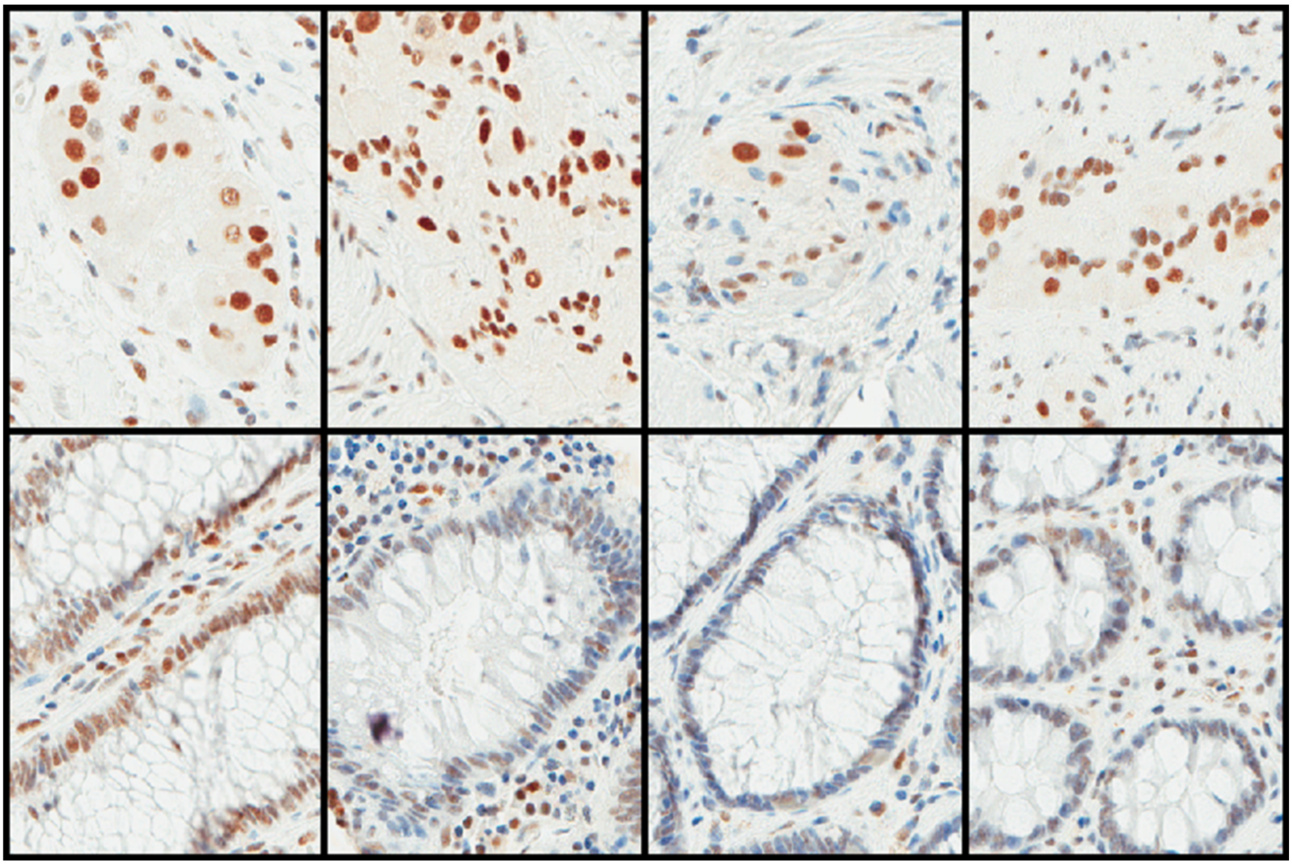

Figure S1: Immunohistochemical staining of selected internal references. Top row: ganglion cells from a single tumor specimen after 6, 24, 48 and 168h fixation. Differences in staining intensity were observed between ganglion cells in a single plexus, however all ganglion cells scored as 3 after 6h fixation relative to the external tonsil reference. Bottom row: crypt epithelium from a single tumor specimen after 6, 24, 48 and 168h fixation. Note the decreasing staining intensity.

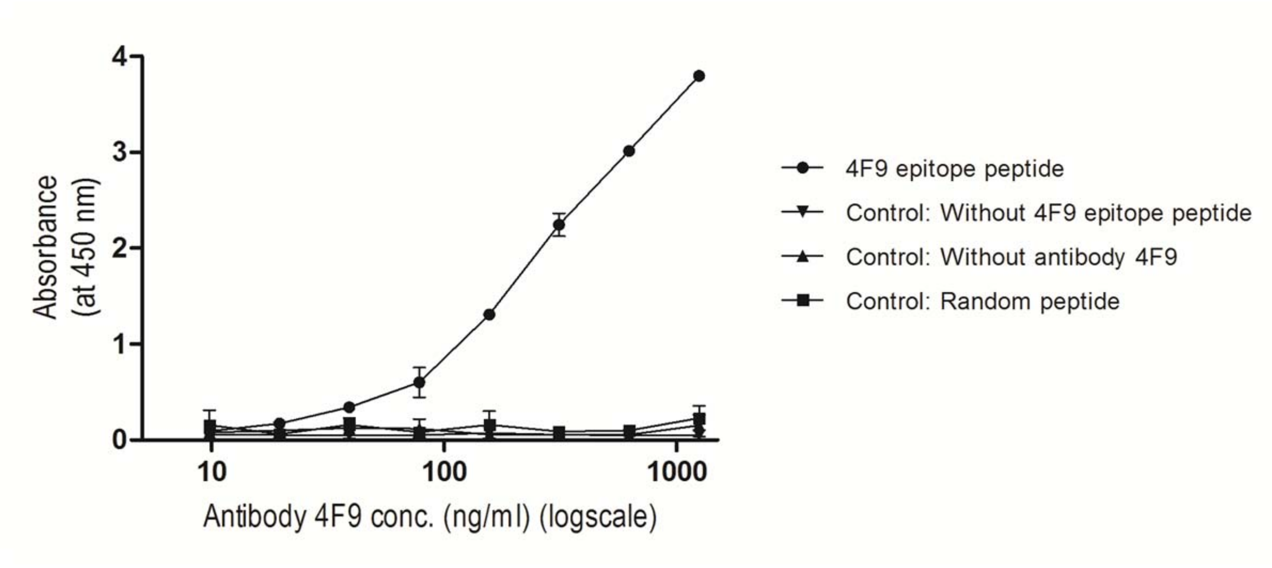

Figure S2: ELISA analysis of antibody 4F9. Wells were coated with either a peptide representing the 4F9 epitope or a random peptide, along with relevant controls. Error bars represent +/-standard deviation on mean absorbance.

Supplementary Tables

Table S1: Staining intensities observed for endothelial cells in paired tonsil specimens fixed for 6 and 30h

Tonsil #	Fixation time (h)	
	6	30
**1**	0, 1, 2	1, 2
**2**	0, 1, 2	0, 1, 2
**3**	0, 1, 2	0, 1, 2
**4**	0, 1, 2	0, 1, 2
**5**	0, 1, 2	1, 2
**6**	1, 2	1, 2

Note: Previous studies with antibody 8F1 have used tonsil tissue as an external control, specifically endothelial cells which were assigned a score of 2 on a scale from 0 to 3 [4]. In the present study, a comparison of paired tonsil specimens fixed for 6 and 30h revealed no significant differences, indicating that tonsil tissue may in fact be suitable as an external control of staining intensities in this particular fixation time interval. Particularly, endothelial cells with a score of 2 were present in all specimens at both fixation times.

Table S2: Staining intensities observed for squamous epithelium in paired tonsil specimens fixed for 6 and 30h

**Tonsil #**	**Fixation time (h)**	
	6	30
**1**	0, 1, 2, 3	0, 1, 2, 3
**2**	0, 1, 2, 3	0, 1, 2, 3
**3**	0, 1, 2, 3	0, 1, 2, 3
**4**	0, 1, 2	0, 1, 2, 3
**5**	0, 1, 2, 3	0, 1, 2, 3
**6**	0, 1, 2, 3	0, 1, 2

Table S3: Staining intensity of internal references in training study relative to a tonsil reference.

Staining intensity	Ganglion Cells (%)	Crypt Epithelium (%)
3	16 (34.8)	-
2 and 3	25 (54.3)	-
2	4 (8.9)	-
1 and 2	1 (2.2)	45 (93.8)
0, 1 and 2	-	3 (6.3)

Table S4: Comparison of *ERCC1* FISH status to IHC-positive and –negative tumors

IHC status	FISH Status		Association between FISH and IHC
	Normal	*ERCC1* gain[a]	
**Negative (0-1)**	50 (42.0)	12 (10.1)	0.03[b]
**Positive (2-3)**	35 (29.4)	22 (18.5)	

[a] ERCC1/CEN-2 ratio ≥ 1.5

[b] p-value from fisher's exact test.

Supplementary Reference List

1. Smith,D.H. *et al.* An explorative analysis of ERCC1-19q13 copy number aberrations in a chemonaive stage III colorectal cancer cohort. *BMC cancer* **13**, 489 (2013).

2. Friboulet,L. *et al.* ERCC1 Isoform Expression and DNA Repair in Non–Small-Cell Lung Cancer. *New England Journal of Medicine* **368**, 1101-1110 (2013).

3. Smith,D.H. *et al.* An explorative analysis of ERCC1/ERCC4 copy number alterations in a chemonaive stage III colorectal cancer patient cohort. *Proceedings of the 104th Annual Meeting of the American Association for Cancer Research* **73**, Abstract 1912 (2013).

4. Olaussen,K.A. *et al.* DNA repair by ERCC1 in non-small-cell lung cancer and cisplatin-based adjuvant chemotherapy. *New England Journal of Medicine* **355**, 983-991 (2006).
